# Design, Construction and Validation of an Electrical Impedance Probe with Contact Force and Temperature Sensors Suitable for *in-vivo* Measurements

**DOI:** 10.1038/s41598-018-33221-4

**Published:** 2018-10-04

**Authors:** Albert Ruiz-Vargas, Antoni Ivorra, John William Arkwright

**Affiliations:** 1 The Medical Device Research Institute, College of Science and Engineering, Flinders University, Adelaide, Australia; 2 Department of Information and Communication Technologies, Universitat Pompeu Fabra, Barcelona, Spain; 3 Serra Húnter Programme, Universitat Pompeu Fabra, Barcelona, Spain

## Abstract

Bioimpedance spectroscopy measurements can be used for tissue characterization. These measurements can be performed in soft tissues by direct contact of a non-invasive probe consisting of two or four electrodes. The amount of force applied by users can be quite different, and the measurements can vary as a result. To compensate for this, we have built an electrical impedance probe (diameter 3.2 mm) with fibre optic contact-force and temperature sensors built in it. The different sensors of the probe were tested individually. The errors in magnitude and phase angle of the probe are <0.9% and <4°, respectively, for a 0.9% NaCl solution. The linear dynamic range of the force sensor was from 0 to 100 grams. An *ex-vivo* experiment on a section of proximal colon from a guinea-pig was performed. Twenty bioimpedance measurements were taken in a frequency range of 5 kHz to 1 MHz, while simultaneously recording the force applied. For an increase in contact pressure applied to tissue from 0 to 15.4 kPa, the maximum change in resistivity was 33% at 5 kHz and the minimum was 6.6% at 142 kHz. The probe is small enough to be introduced via the instrument port of an endoscope.

## Introduction

Bioimpedance spectroscopy measurements have shown their potential to create diagnostic and monitoring tools within the clinical community^1,2^. Bioimpedance measurements can be used to characterize biological tissue, detect and monitor physiological events. They are produced by exciting the tissue with an ac current and measuring the voltage drop between electrodes^3^. Biological tissues have a frequency-dependency behaviour: at low frequency (<1 kHz) currents flow through extracellular body fluids while at high frequencies (>1 MHz) currents flow through cell membranes and intracellular fluid^4,5^. As damage occurs in tissue, its structure changes and so do the current paths. For example, cellular edema will decrease volume in the extra-cellular space and that will cause an increase of the impedance magnitude at low frequencies^6^.

Bioimpedance measurements can be performed by inserting needle electrodes into the tissue or by surface electrodes in contact with the tissue^1^. Using needles, there is a risk of damaging the tissue. Contact electrodes avoid damaging the tissue. However, in *in-vivo* studies it has been shown that contact electrodes produce measurements with big variability^7–9^. That is, measurements with contact electrodes are much less repetitive than those with needle electrodes. It is thought that the main reason for such lack of repeatability is variability in the applied contact-force. This force causes an increase in the pressure within the tissue which pushes away the extra-cellular fluids hence causing an increase in the impedance^10^. In addition, if the sample is sandwiched between the probe and an insulating surface (as it is the case in the present study), this force compresses the tissue sample and reduces its thickness hence causing a further increase in the impedance.

The variability of readings of a small probe (diameter 3.2 mm) due to the force applied has been demonstrated to be significant in *in-vitro* studies^8,9^. In these studies, the applied force was recorded by a weighing scale and the contact pressure was calculated as the ratio between the force and the contact area of the probe. Gonzalez-Correa *et al*.^9^ showed an increase up to 80% on resistivity readings on human gastric tissue for a contact pressure range of 1 kPa to 50 kPa. Lundin *et al*.^8^ reported the resistivity readings taken in a rabbit oesophagus when applying light and hard force manually, showing a higher resistivity when hard force was applied.

Khestar^11^ studied the effect of the probe size on the variability of readings. He concluded that the smaller the probe, the bigger the variability. This is because larger probe size avoids the expulsion of fluid below the tip^11^. However, the size of the probe is critical for minimally invasive clinical procedures when using an endoscope. Therefore, there is a need for a small device capable of monitoring the applied force *in vivo* during the measurement.

The main aim is to avoid the variability problem stated above by embedding a force sensor in the tip of the probe. In general solid-state pressure sensors are not adequate for this application because of their size. A further issue for electrical sensors for these sort of applications is that the long leads are susceptible to electrical noise and they impose additional hurdles regarding electrical safety. For these reasons, we have explored an alternative option based on fibre optics sensing technology. Similar approach, using fibre optic sensing technology, was used before on catheters for cardiac radio frequency ablation to prevent cardiac damage during ablation^12^.

Fibre optic techniques are particularly suitable for biomedical applications due to the small size, high flexibility and immunity to electromagnetic interference^13–16^. In particular, fiber Bragg gratings (FBGs) have the advantage of high sensitivity and stability and have been used to measure pressure, strain and temperature^13,17–20^ making them highly suited for this application.

This paper describes the design, implementation and validation of what appears to be the first probe for bioimpedance measurement with an integrated fibre optic contact-force sensor. A calibration method for the contact-force and temperature is presented in the manuscript. Finally, a test of the probe is reported using a section of excised proximal colon from a guinea pig.

## Bioimpedance Measurement Basis

Bioimpedance is a short term for referring to the electrical impedance of a biological sample measured across electrodes. Electrical impedance is the ratio of voltage and current phasors for a given frequency. The impedance value, Z, is a complex number composed of a magnitude (ratio of voltage and current magnitudes) and a phase angle (difference of voltage and current phase angles). It is often indicated as:

1 $$Z=R+jX$$

where R (units in Ω) is the resistance, X (units in Ω) is the reactance and j is the unit imaginary number.

Bioimpedance measurements can be performed with a pair of electrodes. The same pair is used both for injecting the current and for measuring the voltage drop. This method is known as two-electrode method. This method, however, causes very significant errors when the bioimpedance of soft tissues is measured. This is because the electrode-tissue interface impedance is substantial and, since it is in series with the impedance of interest, adds to the measurements. This significantly perturbs the bioimpedance measurements, especially, at low frequencies^21^.

**Figure 1:**
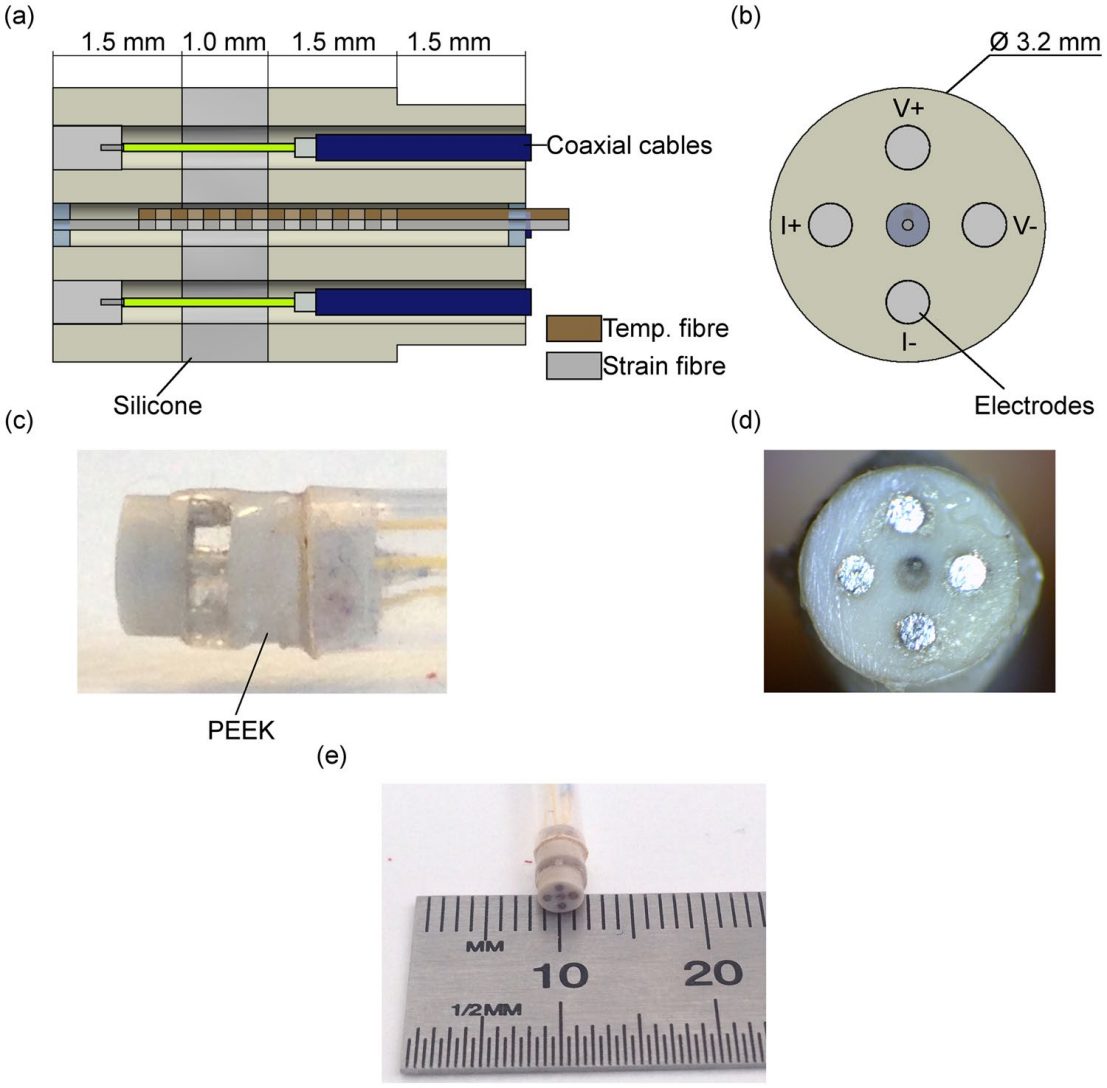
Drawings and images of electrical impedance probe: (**a**) Lateral cross section of the probe design with coaxial cables, electrodes and fibres assembled. (**b**) Front view of the probe design. (**c**) Lateral view and (**d**) front view of the pencil probe. (**e**) Device end-effector after assembly.

An alternative method known as four-electrode method is used to overcome this. The current is injected through two electrodes (I+ and I−) and the voltage drop is measured across the other two electrodes (V+ and V−), see Fig. 1(b). This method ideally cancels the impact of electrode-electrolyte interface impedance on the measurements. However, parasitic impedances at the wires and the instrumentation system cause additional sources of errors that constrain the highest frequency that the system can measure^21^.

Once the signals (current and voltage) are digitized, a signal processing operation “coherent demodulation” is employed to obtain the resistance and reactance of the bioimpedance measurement at a certain frequency. Resistivity values can be obtained by employing a scaling factor (cell constant), which depends on the geometry of the electrode setup. The process to calculate the scale factor is presented in the impedance calibration subsection.

## Fibre Bragg Gratings

Fibre Bragg Gratings (FBGs) have been used in medical applications with great success over the past few decades^22^. Our research group has been working in this area for over 10 years for different medical indications such as *in-vivo* manometry interface pressure measurement^23–26^. FBGs consist of closely spaced variations in refractive index written into the core of photosensitive optical fibre by UV light. The periodicity of the structure defines the specific wavelength of light that is reflected from the grating, known as Bragg wavelength (equation 2). When guided light from a broadband source is launched into the fibre core, these sensing elements reflect the light back at specific wavelength that can be detected by an optical spectrometer.

2 $${\lambda }_{B}=2{n}_{{e}_{ff}}\times \Lambda $$

where Λ is the pitch of the grating, n_eff_ is the effective refractive index of the propagating light, and λ_B_ is the Bragg wavelength. Perturbations such as strain or temperature change the pitch or the refractive index of the grating respectively, hence shifting the Bragg wavelength. The following equation (equation 3) represents the changes in wavelength by strain and temperature:

3 $$\frac{{\rm{\Delta }}\lambda }{{\lambda }_{0}}=k\,\ast \,\varepsilon +{\alpha }_{\delta }\,\ast \,{\rm{\Delta }}T$$

where k is gage factor, ε is the strain, α_δ_ is the change of the refraction index, and ΔT is the temperature change. The first term describes the wavelength shift induced to the fibre due to mechanical and thermal strain and the second one describes the change of refraction index caused by temperature. Equation 4 is more detailed and takes into account the thermal expansion coefficient of package, α_pk_, when the grating is fixed by both extremes or thermal expansion coefficient of glass, α_glass_, when the grating is only fixed by one side:

4 $$\frac{{\rm{\Delta }}\lambda }{{\lambda }_{0}}=(1-{p}_{e})\,\ast \,{\varepsilon }_{m}+((1-{p}_{e})\,\ast \,({\alpha }_{pk/glass})+\eta )\,\ast \,{\rm{\Delta }}T$$

where:

Δ*λ*: Wavelength shift.

*λ*_o_: Initial wavelength.

*p*_*e*_: Photo-elastic coefficient of silica, (*p*_*e*_ = 0.022).

*ε*_*m*_: Mechanical strain.

*α*_*pk*_: Thermal expansion coefficient of package.

*α*_*glass*_: Thermal expansion coefficient of glass, *α*_*glass*_ = 0.55 × 10^−6^/°C.

*η*: Thermo-optic coefficient of the refractive index, *η* = 8.6 × 10^−6^/°C.

Δ*T*: Temperature change in °C.

Different techniques are employed to compensate the temperature effect on the grating that measures strain^27,28^. In the device presented here, a second reference grating that was only connected to the package at one end was used to compensate the temperature and package effect due to the thermal expansion.

## Materials and Methods

### Design and Construction

Figure 1 shows the tip of the developed four-electrode probe with an integrated temperature compensated fibre optic force sensor. The tip is composed of three parts; two Polyether Ether Ketone (PEEK) (Ketron PEEK 1000, Dotmar Plastic Solutions, Adelaide, South Australia, Australia) parts and a compliant silicone pad. The design (see Fig. 1) consists of 5 orifices; four holes for the electrodes spread and separated 0.9 mm from the axis-centre, and one (in the centre) that holds two optical fibres containing draw tower gratings (DTG, FBGS, Geel, Belgium). The finish tip was 3.2 mm in diameter which makes it suitable for use in endoscope port. Polyether Ether Ketone (PEEK) was used for its biocompatibility and good mechanical properties. The two PEEK parts of the tip were cnc-machined in the Australian National Micro-Fabrication (ANFF-SA) facilities.

The silicone part that acts as a compliant layer was formed in a mould made out of 3 pieces of acrylic sheets. In order to clean and activate the PEEK’s surface to promote bonding of the silicone part, the two PEEK parts were placed in an air plasma at <1 mbar with approximately air flow of 5 sccm per minute, prior to assembly. The two parts were then aligned and separated by pins and introduced into the acrylic mould. Silicone (Sylgard 184 Silicone Elastomer Kit, Down Corning, Midland, Michigan, USA) was poured into the mould and left in a vacuum to degas the silicone. At the end, the tip was baked for an hour at 80° Celsius.

The two optical fibres containing the draw tower gratings were passed through the hole and then clamped and placed under tension using a linear translation stage. The two gratings were positioned next to each other and one of the fibres was broken off close to the distal end of the grating (this grating was used as the reference grating). Both fibres were glued with UV light glue (EPO-TEK OG198-55, Epoxy Technology, Billerica, Massachusetts, USA) at the proximal end of the tip, and the sensing fibre was glued to the distal end of the tip (this fibre contained the contact-force sensing grating).

Electrodes were made of platinum wires (0.5 mm in diameter and 0.7 mm in length). These were then pressed into the tip and held in place due to an interference fit with the core of 1.95 m long and 0.3 mm in diameter coaxial cables of (Temp-Flex 50MCX-37, Molex, Lisle, Illinois, USA). UV glue (EPO-TEK OG198-55) then was applied on the surface of the tip to seal any cavities. The parts were then cured for another hour in the oven at 80 degrees. Once cured, the electrodes were filed and polished to form the tip.

Finally, optical fibres and coaxial cables were passed through a 2.8 mm inner diameter and 3.3 mm outer diameter PolyTetraFlouroethelene tube (PTFE AWG 10 T, Adtech Polymer Engineering Ltd, Aston Down East, Stroud, UK). Then, the PTFE tube was attached to the end of the tip with UV glue, see Fig. 1(e). DB-9 connector and E-2000 connectors were used for the coaxial cables and optical fibers respectively.

### Equipment

**Figure 2:**
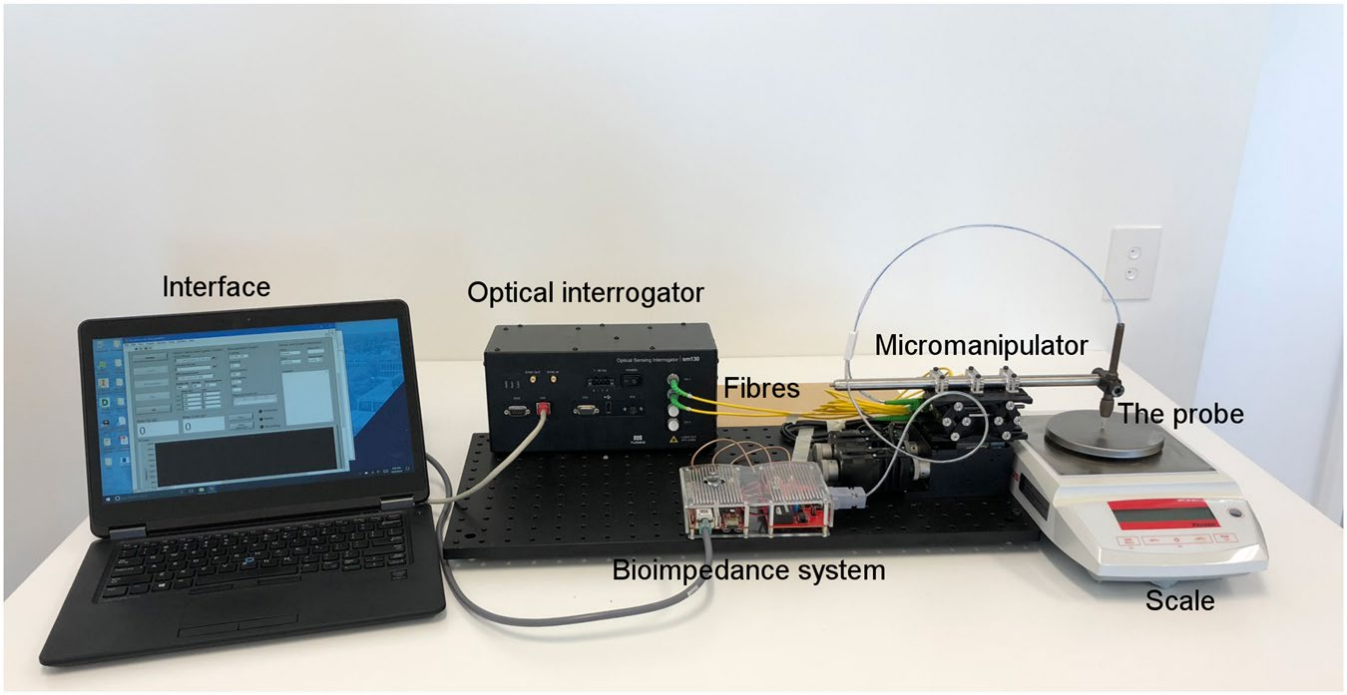
Experimental set-up used during the bioimpedance measurements on the tissue.

Figure 2 shows a picture of the whole measurement system and experimental set-up. The tip sensor was connected to a custom-made bioimpedance measurement system (BIS) described elsewhere^29^ and a Micron Optics sm130 interrogator (MOI) (sm130, Micron Optics, Atlanta, Georgia, USA).

The BIS is capable of measuring complex impedance from 10 Hz to 1 MHz in four-electrode configurations. An alternating current with a maximum amplitude of 10 μA, which is below the maximum auxiliary current allowable by safety standard IEC 60601-1 for any electromedical device, was applied between two adjacent electrodes at each predetermined measuring frequency, and simultaneously the voltage drop between the other two adjacent electrodes was measured^29^.

The MOI system interrogates fibres from 1510 nm to 1590 nm wavelength at 1 kHz. To reduce the noise of the readings 100 samples were used to average a single sample, decreasing the effective sampling rate down to 10 Hz.

A force scale (Pioneer PA143, Ohaus, Parsippany, New Jersey, USA) was used to calibrate the device and compare with the data from the force sensor.

Output data from the BIS, the MOI and the scale were displayed and recorded in real time using a custom software application built in LabVIEW (version 2015 sp1, National Instruments, Austin, Texas, USA).

### Impedance calibration

Two agar-agar models prepared with 0.9% (0.150 M) and 0.09% (0.0150 M) NaCl saline solutions were used to calibrate the impedance probe and determine the spectrum range of the whole impedance measurement system. The conductivities of these two samples (1.440 S/m and 0.144 S/m) approximates the range of conductivities of gastrointestinal tissue^30^. The probe was positioned on each agar-agar model and the impedance data at 14 different frequencies in the frequency range of 1 kHz to 1 MHz, separated logarithmically, were obtained. A hundred measurements were taken to generate average values of the impedance magnitude and phase angle at each frequency.

From those measurements, we evaluated the useful frequency range and calculated the cell constant of the electrode configuration. The cell constant is a scale factor for converting the resistance values (R, units: Ω) into resistivity (ρ, units: Ω.m), and is calculated according to Equation 5.

5 $$K=\frac{R}{\rho }[{m}^{-1}]$$

In addition, the cell constant was also computed through a simulation in ANSYS Maxwell (ANSYS Electronics Desktop, ANSYS Inc, Canonsburg, Pennsylvania, USA). The model consisted of a cylindrical PEEK part (length = 2 mm, outer diameter = 3.2 mm) containing four platinum electrodes (length = 2 mm, outer diameter = 0.5 mm, spread and separated 0.9 mm from the axis-centre), a bed surface and a saline content (between the tip and the bed surface). The material properties for the PEEK part (plastic: ε_r_ = 3.2), electrodes (platinum: ε_r_ = 1, σ = 9.3 Ms/m) and bed surface (glass: ε_r_ = 5.5) were obtained from the Material Library provided in ANSYS Electronics. The cell constant was analysed as a function of distance between the tip and the bed surface. In addition, the sensitivity depth of the probe was analysed and calculated using the method presented by Gonzalez-Correa *et al*.^31^.

### Contact-force calibration and validation

A set-up was prepared to characterize the contact force sensor. The probe was vertically hold in a hollow pin vice that was attached to a vertical translation stage. The probe was then pushed down onto a weight scale, wavelengths were recorded at 0 grams and 100 grams to calculate the change in wavelength per gram. After the calibration of the force sensor, the probe was pushed against the balance/scale in increments of 0.01 mm until it reached around 70 grams, and then was retracted in increments of 0.01 mm until it reached 0 grams. The data from the tip was acquired at 10 Hz while the acquisition frequency of the scale was fixed at 11.7 Hz.

Two additional experiments were performed to validate the contact force sensor using agar models which have similar elastic properties as soft tissue^32^: Firstly we used the same set up as for calibration, but in this case, we increased the force faster and at different increments, Secondly the probe was handled manually to give different peak intensities.

### Temperature compensation

As mentioned above, FBGs are temperature dependent, and so to remove the temperature artefact we included two FBGs one that was sensitive to both temperature and force, and the other to just temperature alone. The configuration was tested in an oven using a separate thermocouple (U1186A, Keysight Technologies, Santa Rosa, California, USA) to track the ambient temperature. The tip sensor was placed inside a hollow plastic tube inside of an oven to reduce air currents and to provide a more uniform heating environment. The temperature was increased from 21 °C to 40 °C and data was recorded from both FBGs until the temperature measured on the thermocouple reached a steady state.

### *Ex Vivo* assay

**Figure 3:**
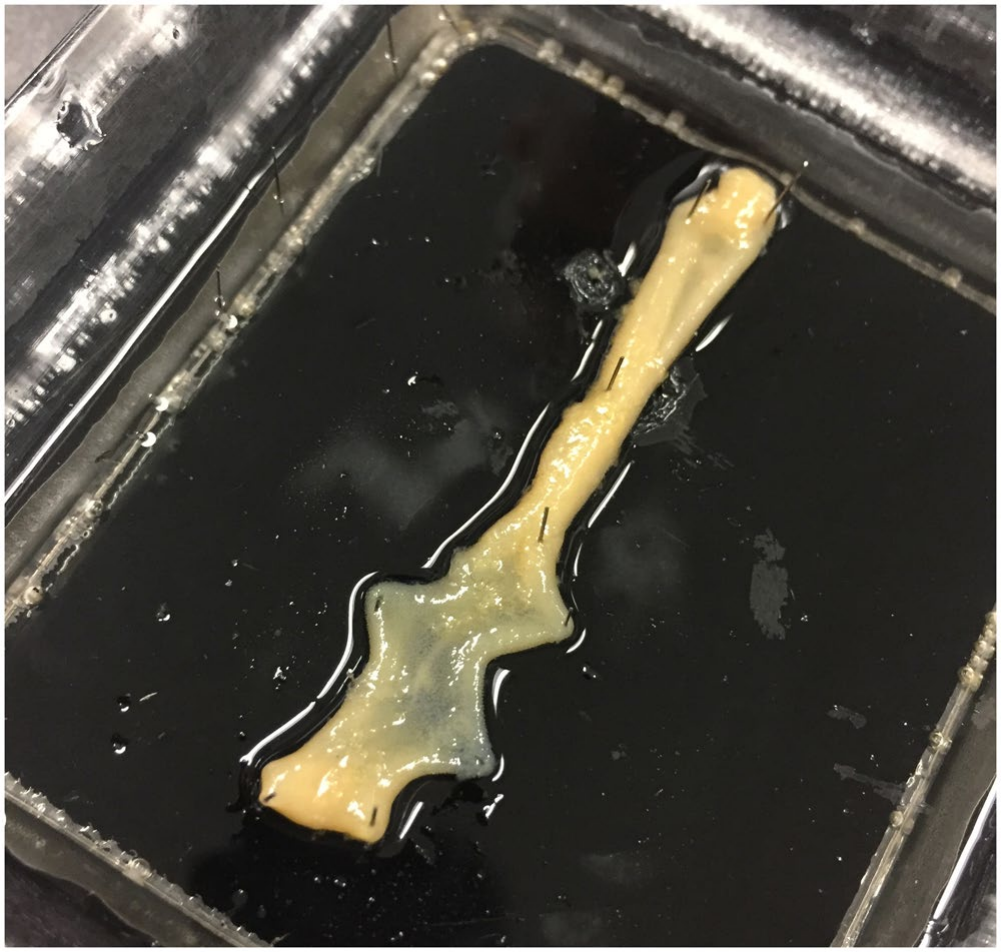
Pinned open proximal colon of a guinea pig used on the experiment.

A segment of a proximal colon from a guinea pig was used. The section was removed from the laboratory animal by methods approved by the Animal Welfare Committee of Flinders University (Animal Ethics Number: 845/12). All methods were performed in accordance with the relevant guidelines and regulations. The removed section of colon was placed into a beaker containing oxygenated Krebs solution (in mM: NaCl, 118; KCl, 4.7; NaH_2_PO_4_, 1.0; NaHCO_3_, 25; MgCl_2_, 1.2; D- Glucose, 11; CaCl_2_, 2.5) and bubbled with 95% O_2_/5% CO_2_. The faecal pellets were gently flushed out of the colonic segment with Krebs solution. Then the segment was cut, opened and fixed with pins onto a tray with a hard silicone surface, Fig. 3.

The preparation was placed on the electronic weight scale, see Fig. 2. The probe was then lowered downwards onto the tissue while the system was recording the force from the weight scale and from the tip, and the bioimpedance measurements. The sample frequency for the force data was 10 Hz and 11.7 Hz for the tip and scale, respectively. Twenty bioimpedance measurement at different frequencies in the frequency range of 5 kHz to 1 MHz were performed at a data acquisition rate of 1.5 Hz, which was the maximum sample frequency achievable by the system under these conditions.

## Results

### Impedance calibration

**Figure 4:**
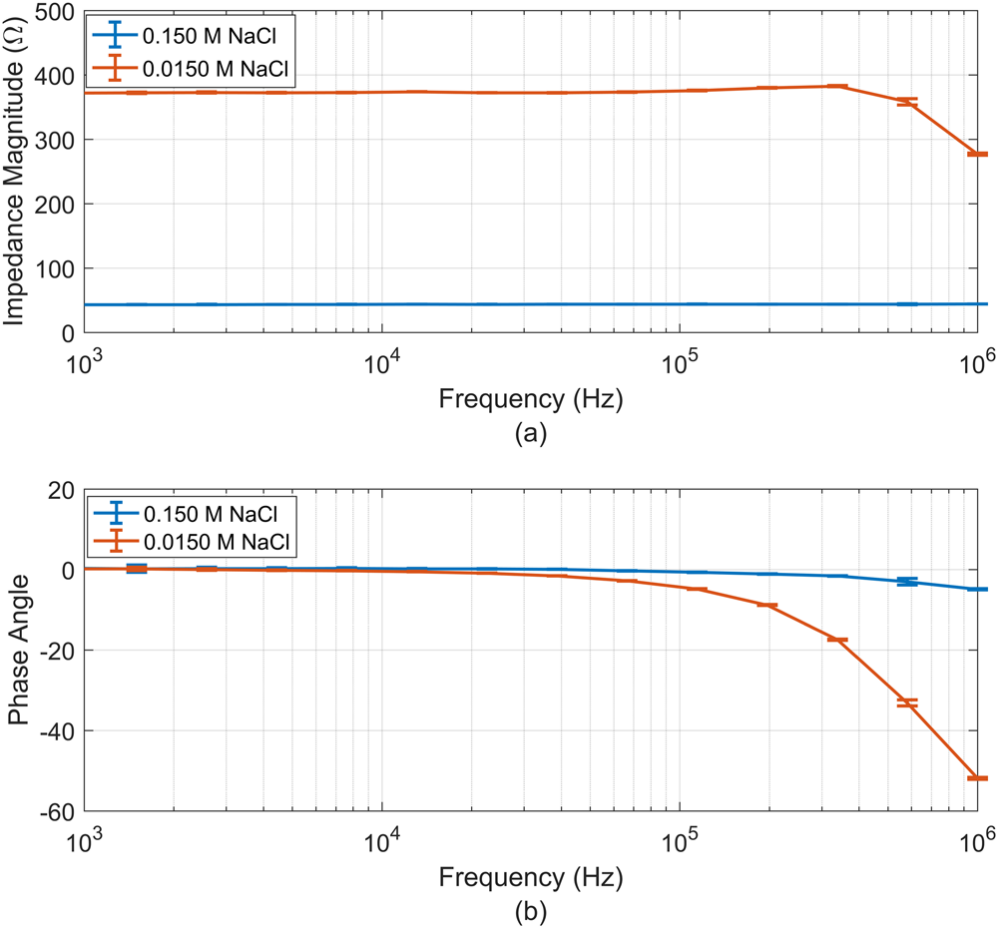
Impedance measurement of the agar-agar samples made from 0.15 M and 0.015 M NaCl: (**a**) Impedance magnitude and (**b**) Phase angle along the frequency range 1 kHz to 1 MHz.

Figure 4 shows the impedance magnitude and phase angle of the probe for the spectrum range 1 kHz to 1 MHz. Saline solutions exhibit no dielectric dispersion at frequencies up to 1 MHz, so impedance magnitude and phase angle should be constant. From the results it can be concluded that for conductivities similar to 0.150 M NaCl the error in phase is <4° and the relative error in magnitude is <0.9% from 1 kHz to 1 MHz. Whereas for conductivities similar to 0.0150 M NaCl, the error in phase and the relative error in magnitude increased to 50° and 25%, respectively, due to the effect of the stray capacitances of the probe and BIS system. When the systematic error is not significant the scaling factor of the probe and BIS system is 53.8 m^−1^. This cell constant value was calculated averaging the measured resistance values for the frequency range 1 kHz to 200 kHz and assuming that the resistivity of the 0.09%NaCl Agar-Agar model is 0.144 S/m. Mean and standard deviation of resistance for the interval were 373.61 Ω and 1.12 Ω, respectively.

The simulated cell constant was 78 m^−1^ for sample thicknesses larger than 3 mm. However, the cell constant was 6% and 19% larger if the distance between the probe surface and the bed surface are 2 mm and 1.5 mm respectively. Ninety percent of the information of our probe comes from the region above a depth of 0.79 mm. These results of depth penetration are similar to those by Gonzalez-Correa *et al*.^31^; they reported that their probe had a depth of 0.77 mm.

### Contact force calibration

**Figure 5:**
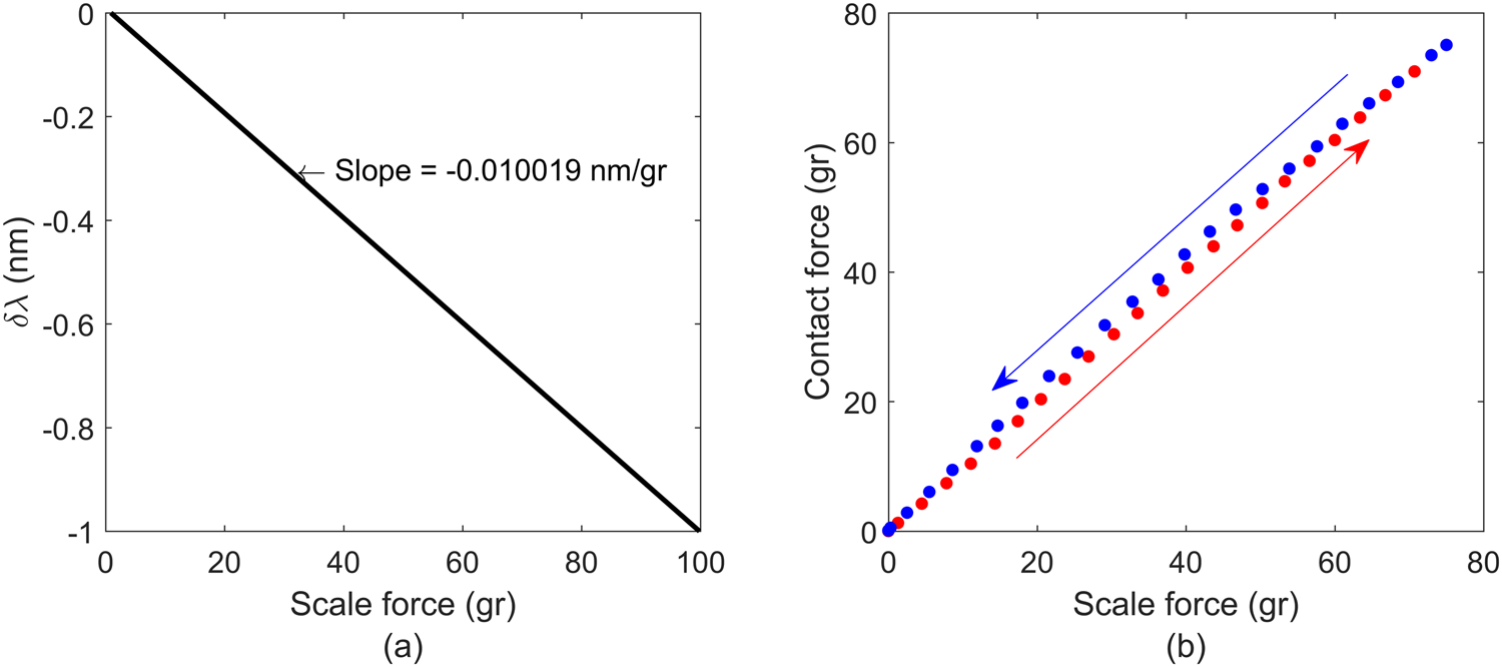
(**a**) Points used for the calibration of the contact force. (**b**) Points measured during a test while adding force and then realising it.

Figure 5(a) shows the wavelength change as a function of contact force measured during calibration of the force sensor, and Fig. 5(b) shows the calibrated output from the sensor using the slope calculated from Fig. 5(a). A slight hysteresis was observed between loading and unloading of the force sensor with a maximum difference of 2 grams. For the purposes of this work the sensor was assumed to be linear over the region of interest.

**Figure 6:**
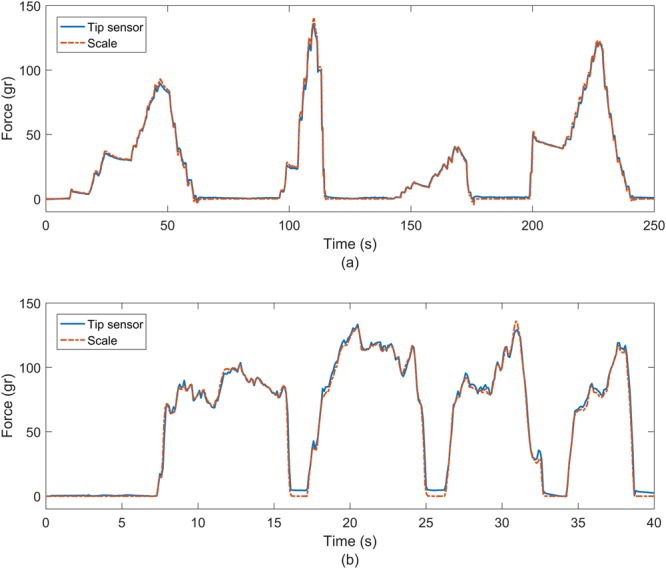
Force test done on an agar-agar model. (**a**) With a micromanipulator pushing downwards, and (**b**) Handled manually.

Figure 6 shows two graphs of two different recordings of the tip sensor on the agar-agar model. Figure 6(a) corresponds to the data obtained by loading the tip sensor with a micromanipulator whereas Fig. 6(b) corresponds to manual loading. Small differences of 1 to 2 grams are observed between the tip sensor and the weight scale signals when the force is applied.

### Temperature compensation

**Figure 7:**
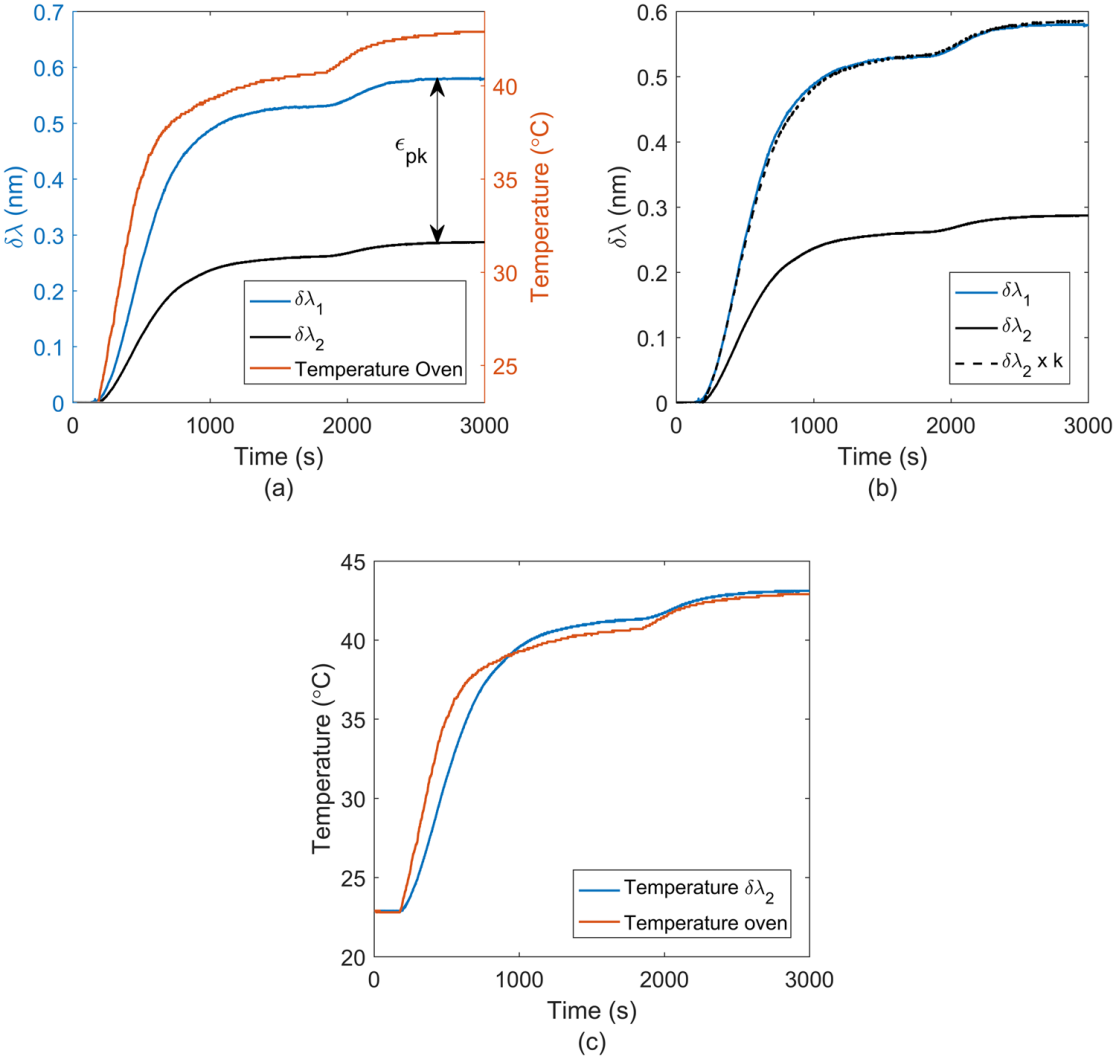
Temperature compensation: (**a**) Wavelength shift for the force sensor (δλ_1_) and temperature sensor (δλ_2_), and the temperature profile during the experiment, (**b**) Wavelength shift (δλ_1_) and (δλ_2_), and a comparison of the wavelength shift of (δλ_1_) and (δλ_2_*k). (**c**) Temperature increment from the thermocouple and the reference grating (δλ_2_).

Figure 7 displays the results obtained from the temperature compensation test. The graph on the left side shows the wavelength shift for the contact-force sensor fibre (δλ_1_), the temperature sensor fibre (δλ_2_), and the temperature profile measured from a thermocouple (accuracy ±1.1 °C and temperature range −20 °C to 200 °C) next to the tip sensor. Wavelength shift is dependent on temperature and strain (see equation 3), wavelength of fibre 1 is susceptible to temperature and strain whereas wavelength shift of fibre 2 is only to temperature. The difference wavelength shift between fibre 1 and fibre 2 is due to the strain induced from the thermal expansion of the package (ε_pk_), see Fig. 7(a).

It is worth noticing, that there is a delay on the heat transfer to the fibre. The thermocouple reacted to the change in temperature before the contact-force sensor due to the thermal insulation of the tip sensor packaging.

Figure 7(b) shows the wavelength shift of both optical fibres 1 and 2 (δλ_1_ and δλ_2_) and compares the wavelength shift of fibre 1 with wavelength shift of fibre 2 multiplied by a constant (k). The k is the ratio of wavelength shift of fibre 1 and 2 (k = 2.038), when both fibres reach steady state at specific temperature and there is zero mechanical strain. The difference between δλ_1_ and δλ_2_ multiplied by the k factor due to temperature effect is very small. The k factor was also measured multiple times 2.050, 2.034 and 2.017 before and after the experiment (mean = 2.036 and std = 0.012).

Figure 7(c) compares the temperature from the thermocouple and the temperature from the reference grating obtained from Equation 3. The difference in temperature between thermocouple and reference grating was less than 1 °C degree for steady state.

### *Ex Vivo* assay

**Figure 8:**
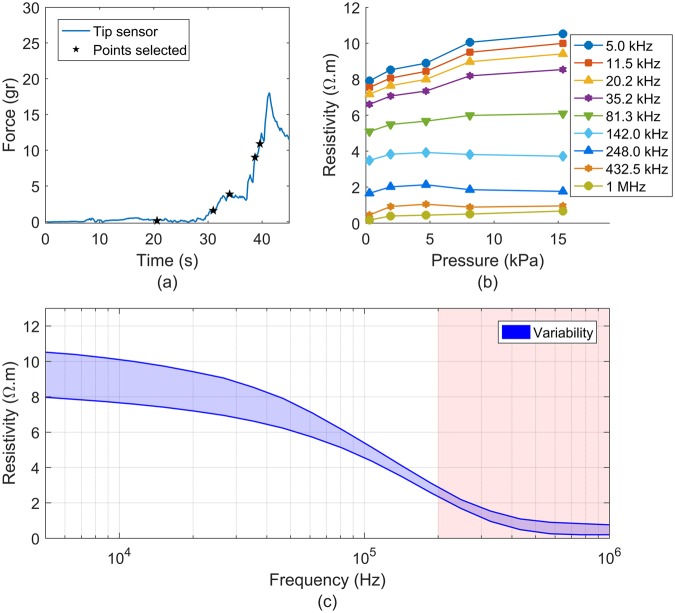
Results of the *ex vivo* experiment: (**a**) Force values in grams obtained during a recording. Star points in (**a**) are the points selected for representing the changes in impedance versus contact pressure. (**b**) Resistivity values in the frequency range of 5 kHz to 1 MHz at different pressures. (**c**) Variability of resistivity measurements along the frequency range 5 kHz to 1 MHz during the measurement shown in (**a**), note that the shaded area from 200 kHz to 1 MHz indicates at which frequency data is unreliable according to the validation presented above.

The results of the *ex vivo* experiment are presented in Fig. 8. Figure 8(a) shows the force measured using the fibre optic force sensor, and the data force points (data force points were converted to contact pressure dividing by distal surface of the tip; 8.0425 mm^2^) values used to generate Fig. 8(b). Points were strategically selected in order to overcome the mismatch of sample frequencies of pressure and impedance. We made sure that at least the three previous pressure points of the selected resistivity points varied by less than 1gram.

Figure 8(b) compares the resistivity values (which were obtained by dividing the real part of the impedance, that is, the resistance, by the K cell constant) at different contact pressure points indicated in Figure 8(a) (0.28, 1.92, 4.7, 8.12 and 15.38 kPa) for frequencies of 5, 11.5, 20.2, 35.2, 81.3, 142, 248, 432.5 kHz and 1 MHz. However, only frequencies from 5 to 142 kHz were considered since are within the viable frequency range of the BIS probe and are close to the frequencies used in a study done by Gonzalez-Correa *et al*.^9^. The resistivity readings, shown in Fig. 8(b), increased considerably with contact pressure for the frequency range of 5 kHz to 81.3 kHz, whereas at 142 kHz the change was much less significant. Figure 8(c) shows the variability of resistivity measurements along the full frequency range during the measurement for the full pressure range (0 to 15.38 kPa). The shaded area indicates the frequencies (>200 kHz) at which the data become unreliable according to the validation performed with the 0.09% NaCl sample. As seen before in Fig. 8(b) a large increase in resistivity can be seen at low frequencies, whereas at high frequencies (~100 kHz) the increase in resistivity decreases.

## Discussion and Conclusions

According to the *in vitro* assays performed here, the developed system should be capable of measuring the impedance of soft living tissues with an error in magnitude of less 0.9% and an error in phase of less 4° in the frequency range from 1 kHz to 1 MHz for conductivities of 1.44 S/m. Parasitic capacitances, which are a source of systematic errors causing a decrease in the measured modulus of the impedance, become significant at high frequencies (>200 kHz) for tissue conductivities of 0.144 S/m. Because it is at high frequencies when the displacement currents through capacitances are relevant. And it is more noticeable for the highest resistivity because those parasitic paths to stray current (instead of going through the sample) become more “preferable” as the impedance of the sample increase. The frequency range is consistent with the frequency range found in similar studies using four-electrode setups^9^. The lower limit of the frequency range is imposed by the sample rate of the BIS measurements to keep up with the sample rate of the fibre optic sensors; the upper limit is mostly imposed by the stray capacitances of the wires within the probe, and to a lesser degree in the BIS system.

The difference between the simulated and the experimental cell constant might be attributed to engineering tolerance in electrode positioning. Inspection under a microscope showed errors of about 250 μm in the fabricated tip. Simulations with such errors were performed, in which cell constant varied from about 50 m^−1^ to about 100 m^−1^.

The FBG sensors proved to be a useful method to assess the contact force and temperature during the bioimpedance measurements. The contact-force sensor was compared with data recorded by an electronic scale. Maximum differences of 1 to 2 gr were observed during the assays.

The temperature test showed that multiplying the reference grating by a constant factor can compensate the temperature effect on the grating under strain. Such standard deviation of k factor (0.012) could induce an error of less than 1 gr, so should not affect significantly the measurements in a short time; however the k factor could change over time due to degradation of silicone. Furthermore, the temperature change from the thermocouple and the fibre are similar, albeit with a slight lag in response of the fibre due to the insulating properties of the tip sensor packaging. This could be improved by increasing the thermal conductivity of the package. The delay in thermalisation was only of the order of 120 s and so should not affect *in-vivo* measurement to any great degree as body temperature is nominally constant and measurements would take longer than this to set up and acquire data. Care may be needed however if irrigation fluid is used as part of an endoscopic procedure.

In this instance, in the *ex vivo* assay results, the maximum change in resistivity due to variations in applied contact pressure was 33% over the range 0 to 15.38 kPa at 5 kHz. The increase in resistivity was found to reduce at higher frequencies being a minimum of 6.6% for at 142 kHz. This increase in apparent resistivity may be explained by the fact that the tissue sample under test gets compressed resulting in both a thickness reduction, which increases the cell constant, and an increase in tissue pressure which causes extracellular liquid migration and an actual increase in tissue resistivity as explained previously in^10^.

During the experiments, we observed that the thickness of the sample always appeared to be above 2 mm (not measured). This implies that, according the simulation results, the maximum increase in impedance we would observe due to a decrease in the sample of the thickness would be in the order of 6%. Therefore, the 33% increase in impedance must be mostly attribute to an actual increase in the resistivity of the tissue.

The above observations highlight the need to control contact pressure to make sure the variability is as small as possible at low frequencies.

The impact of contact force on the bioimpedance measurements is lower when impedance is measured at a sufficiently high frequency, as it can be seen in Fig. 8(c). This can be explained by the fact that the current at high frequencies passes through extracellular and intracellular liquid, and cell membranes^5^. Intracellular liquid is constrained by the cells when a low mechanical compression is applied, unlike the extracellular liquid that is rapidly pushed aside^10^. Therefore, the variability is reduced at high frequencies due to the constrained nature of intracellular liquid and the current’s path at high frequencies.

The resistivity results are consistent with resistivity values obtained in colon of rabbit done by Mulett *et al*. where resistivity values at 19.2 kHz were within the range of 8.7 to 6.6 Ω.m from proximal to distal, respectively^30^. Also, these results qualitatively agree with the ones obtained by Gonzalez *et al*. on columnar gastric tissue of rats^9^. Although, the resistivity values of colon are slightly larger than those for the columnar gastric tissue and much lower than those for the squamous epithelium, these discrepancies can be attributed to both the difference in animal tissue and the difference in structure between gastric columnar and colonic columnar epithelium, which contains more glands surrounded by a vast of secretory and goblet cells than gastric columnar epithelium.

Reporting the resistivity values alone cannot provide details such as structural changes or ischemic damage. However, in this instance, data were reported as the real part of impedance (resistivity) for the sake of simplicity and to be in accordance with previous published data. Other parameters, that can be obtained from the data set – such as phase angle, Cole parameters (α, τ_Z_, R_∞_ and R_0_) and/or distribution of relaxation times (DRT), can provide further information^33–35^. As with the resistivity results, this information will be more consistent if the contact pressure is controlled.

This study set out to develop and evaluate a new medical device for measuring the contact-force applied during bioimpedance measurements on biological samples up to 1 MHz; however, our results indicate that stray capacitances limit the device performance above 200 kHz. The device will allow us to perform a more detail study on the data variability due to the contact pressure effect on the bioimpedance measurements. Its small dimensions make it suitable for endoscopic and colonoscopic procedures.
